# Rare subungual amelanotic melanoma presenting as prolonged swelling and exudation after trauma: case report and literature review

**DOI:** 10.3389/fimmu.2025.1661698

**Published:** 2025-09-26

**Authors:** Lin An, Ziyu Liu, Xiangru Chen, Yuxi Jia

**Affiliations:** Department of Dermatology, China-Japan Union Hospital of Jilin University, Changchun, Jilin, China

**Keywords:** subungual amelanotic melanoma, trauma, post-traumatic immunosuppression, differential diagnosis, immunohistochemistry

## Abstract

**Background:**

Subungual amelanotic melanoma (SAM) poses significant diagnostic challenges due to its rarity and nonspecific clinical manifestations, such as nail dystrophy or indurated plaque.

**Case presentation:**

We present the case of a 60-year-old woman with a three-year history of recurrent serous drainage and persistent pain in her left middle finger following an initial crush injury. Over a period of two years, she underwent three nail avulsion procedures, received systemic antibiotic therapy, and was treated with topical Chinese herbal therapies under a presumptive diagnosis of “chronic onychia following trauma” at a local hospital. Additionally, PET-CT imaging demonstrated localized inflammatory changes without evidence of neoplastic disease. Despite these interventions, the lesion remained refractory to treatment. A thorough reevaluation conducted by our department, incorporating histopathological and immunohistochemical analyses, ultimately confirmed the diagnosis of SAM.

**Conclusions:**

This case underscores the importance of maintaining a high index of suspicion for SAM when evaluating atypical nail lesions. A low threshold for nail biopsy in cases of prolonged swelling and exudation of a single nail is advised. Additionally, prior trauma to the nail may contribute to the development of SAM through post-traumatic immunosuppression and persistent low-grade chronic inflammation. However, the exact role of trauma in the pathogenesis of melanoma remains unclear and requires further investigation.

## Introduction

Amelanotic melanoma (AM) is a rare subtype of melanoma with little or no pigment on visual or histopathologic examination (1). Although AM represents 2% of all malignant melanoma cases, it presents with a greater Breslow depth, higher mitotic rate, more frequent ulceration, higher tumor stage, and lower survival rates than pigmented melanoma (2). Notably, About 25%-33% of subungual melanoma present as amelanotic lesions (3). Subungual amelanotic melanoma (SAM) manifests as a non-pigmented erythematous nodule arising from the nail bed, most commonly affecting the great toe and thumb (4). It often mimic conditions such as paronychias, pyogenic granulomas, hemangiomas, chronic infections, or squamous cell carcinomas (5). Established criteria for the clinical diagnosis of SAM are lacking, often leads to a delay in diagnosis. The combination of its rarity and nonspecific clinical manifestations contributes to an average diagnostic delay. Due to this delay, SAM is typically identified at an advanced stage, resulting in disease progression, and a poor prognosis with challenging treatment options (6).

We report the case of a 60-year-old Chinese woman with SAM on the left middle finger, who experienced a three-year history of recurrent serous drainage and persistent pain of her left middle finger following an initial crush injury. Over a period of two years, she underwent three nail avulsion procedures, received systemic antibiotic therapy, and was treated with topical Chinese herbal therapies under a presumptive diagnosis of “chronic onychia following trauma” at a local hospital. Additionally, PET-CT imaging demonstrated localized inflammatory changes without evidence of neoplastic disease. Despite these interventions, the lesion remained refractory to treatment. A thorough reevaluation conducted by our department, incorporating histopathological and immunohistochemical analyses, ultimately confirmed the diagnosis of SAM The patient’s history of trauma, along with the atypical clinical presentation significantly increase the likelihood of misdiagnosis, further underscoring the the diagnostic challenges inherent in SAM.

## Case presentation

The patient was a 60-year-old Chinese female who presented to our dermatology department with a three-year history of recurrent swelling and exudation of the left middle finger. The condition originated three years earlier when the nail sustained trauma due to a door closure incident, after which it appeared normal initially. Approximately two and a half years prior, purulent discharge began to exude from the nail. The patient had attempted self-management using a topical Chinese herbal formulation known as Yunnan Baiyao for one month, but experienced no significant improvement. Subsequently, she sought care at the Hand and Foot Surgery Department of Hospital A and was diagnosed with chronic onychia following nail trauma. Over a period of two years, she underwent three nail avulsions, was treated with local red light therapy and received systemic antibiotic therapy. Specifically, following the first avulsion, she was prescribed cefuroxime axetil 500 mg twice daily for five days. After the second avulsion, she was treated with moxifloxacin 400 mg once daily for seven days. However, no significant clinical improvement was observed. Following the last avulsion, the nail failed to regrow, and the surgical wound did not heal properly, resulting in a persistent, painless wound on the nail bed of her left middle finger. Seeking further evaluation and treatment, the patient consulted the Hand and Foot Surgery Department at the China-Japan Union Hospital of Jilin University. Onychomycosis was suspected by the attending physician, and the patient was referred to our department for further assessment. During the disease course, the patient denied any systemic symptoms (e.g., fatigue, weight loss) and reported no history of infectious diseases, relevant family history, or personal history of cancer.

**Figure 1:**
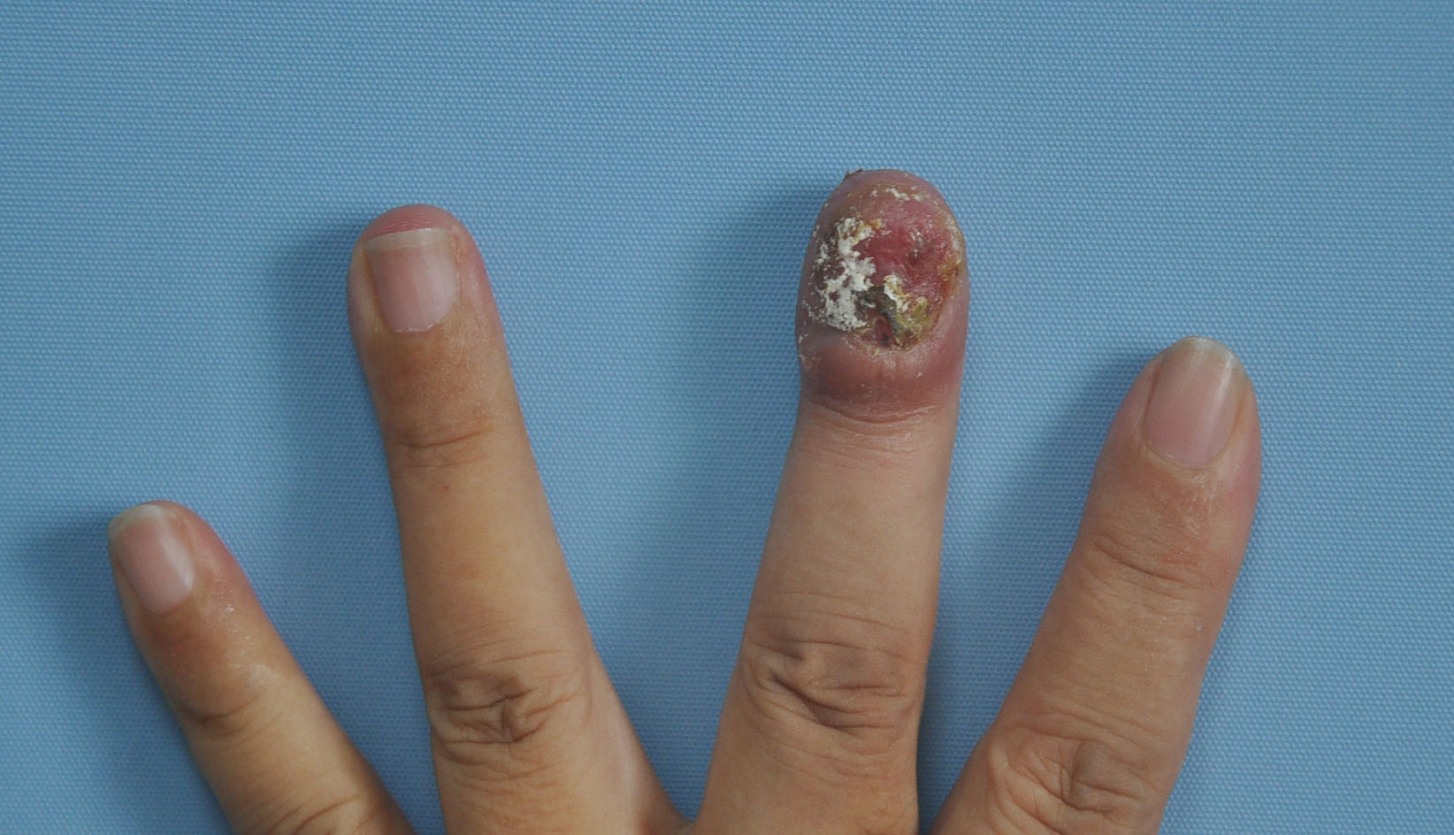
Images of the lesion on the left middle finger. It exhibited complete absence of the nail plate, accompanied by an irregularly swollen nail bed with serous exudation and crusting. And the margins of the skin lesion demonstrated signs of infiltration.

On physical examination, the left middle finger exhibited complete absence of the nail plate, accompanied by an irregularly swollen nail bed with serous exudation and crusting. And the margins of the skin lesion demonstrated signs of infiltration (**Figure 1**). Palpation revealed a moderately firm texture with mild tenderness. Black pigmentation of the adjacent nail fold, known as Hutchinson’s sign, is often considered a diagnostic indicator; however, in this patient, Hutchinson’s sign was negative. No abnormalities were noted in the remaining skin areas upon examination. There was no palpable enlarged superficial lymph nodes throughout the body.

**Figure 2:**
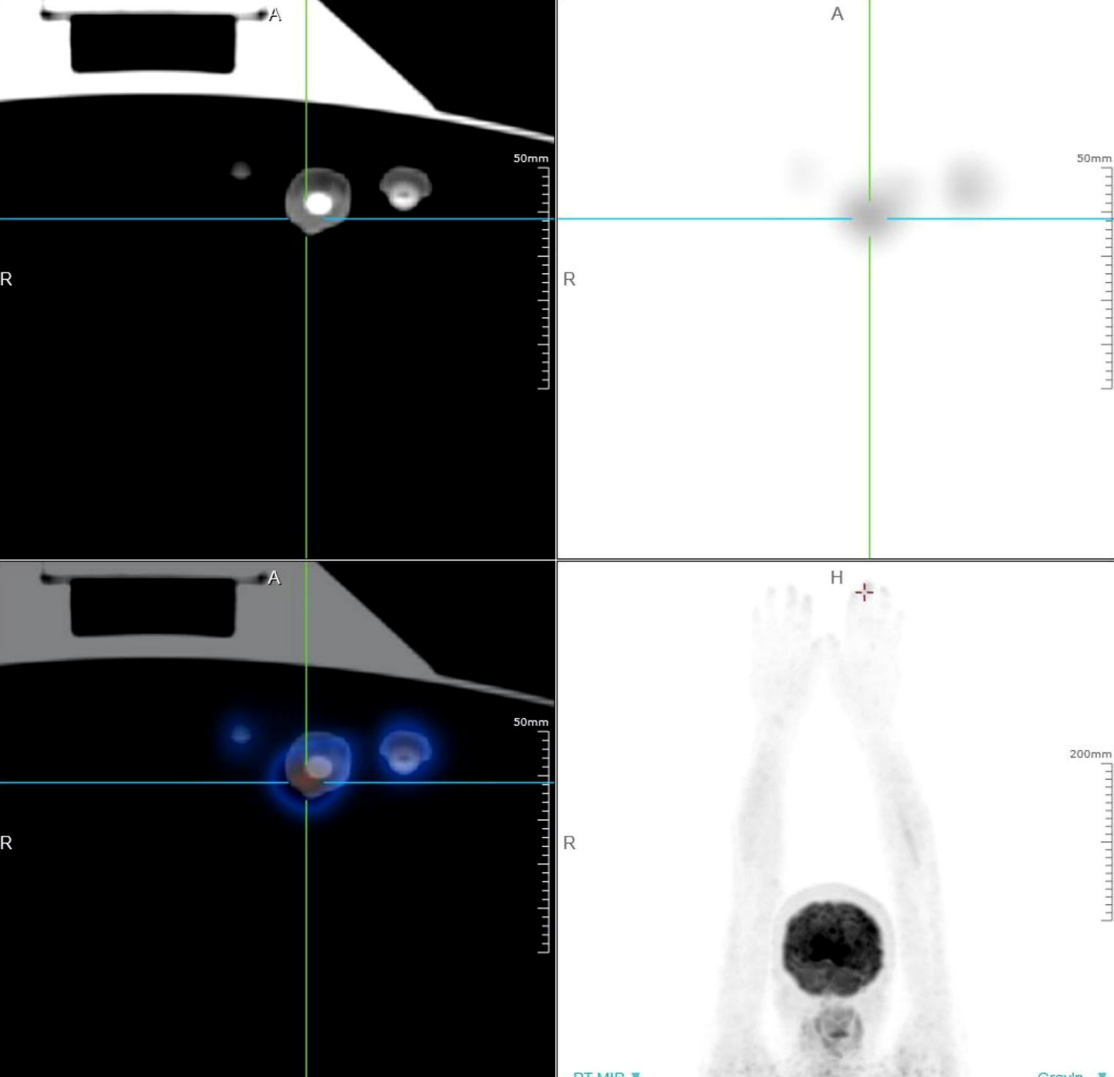
PET-CT scan of the patient with SAM. It demonstrated soft tissue thickening and mild glucose hypermetabolism (SUV max 2.23) around the distal phalanx of the left middle finger, indicative of inflammatory changes.

**Figure 3:**
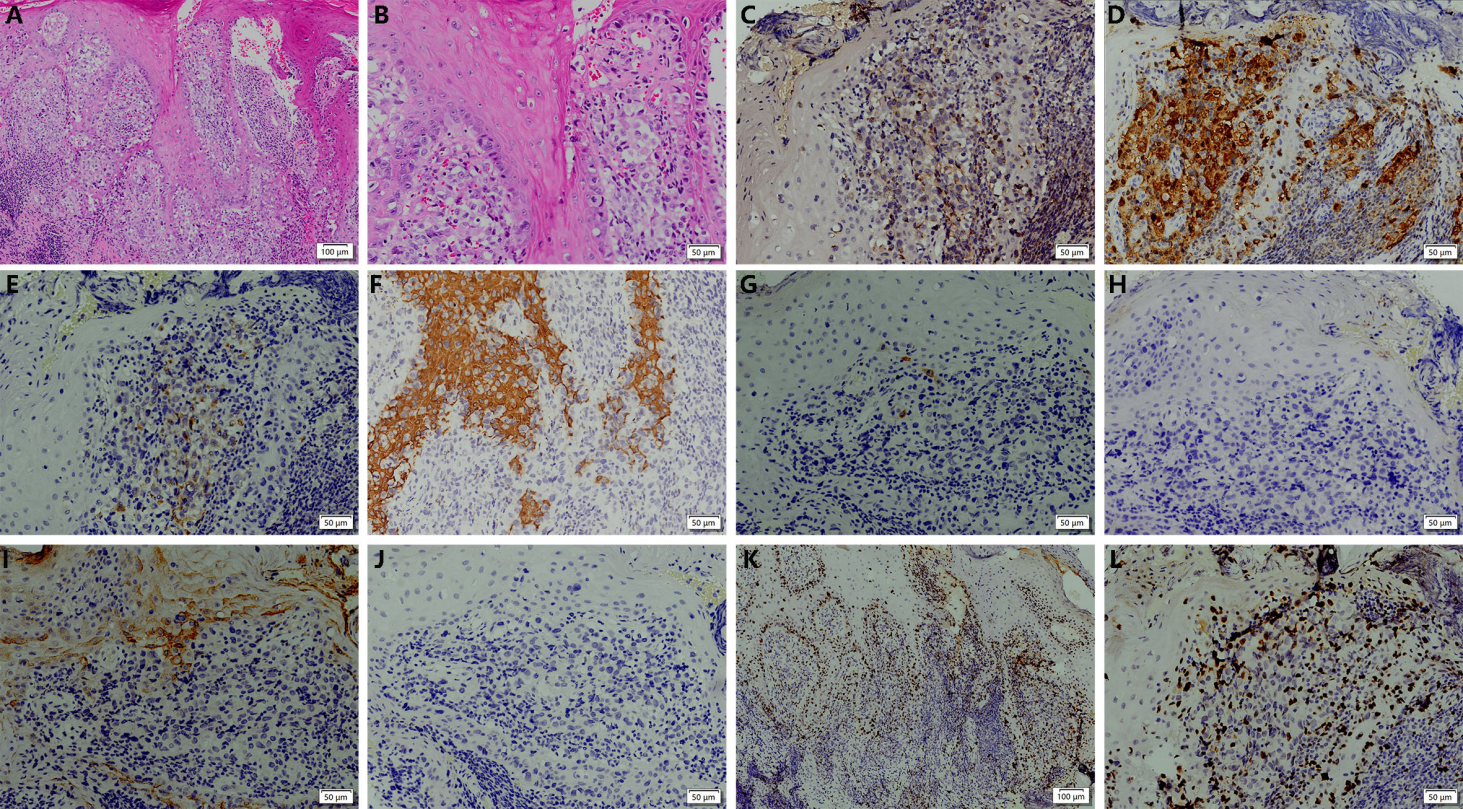
Histological and immunohistochemical examination of the lesion on the left middle finger. **(A)** Histopathological examination revealed the presence of tumor cell nests at the dermoepidermal junction, with no evidence of pigmentation. Additionally, pagetoid spread of tumor cells was identified within the epidermis. **(B)** The tumor cells exhibited marked cellular atypia, intense nuclear staining, and clear cytoplasm. **(C-J)** Immunohistochemical staining for HMB45 (diffuse +), S100 (diffuse +), Melan-A (+), and CK5/6 (–), CD7 (–), CEA (–), EMA (–) and Her-2 (–). **(K, L)** The Ki-67 proliferation index was 20%, consistent with aggressive biological behavior.

We conducted relevant laboratory and auxiliary tests. Fungal microscopy and culture were conducted, which came back negative. Given the potential for skin tumors, we advised the patient to undergo a histopathological examination, but the patient refused. Based on this, we alternatively proposed a Positron Emission Tomography-Computed Tomography (PET-CT) scan as a less invasive diagnostic approach. It demonstrated soft tissue thickening and mild glucose hypermetabolism (SUV max 2.23) around the distal phalanx of the left middle finger, indicative of inflammatory changes (**Figure 2**). However, local and systemic anti-inflammatory treatments have shown no improvement. Pathological examination was recommended to rule out neoplastic recurrence. Subsequently, a nail bed biopsy was performed and histopathological examination revealed nests of tumor cells at the dermoepidermal junction, exhibiting marked atypia, deep nuclear staining, and transparent cytoplasm. Additionally, Pagetoid spreading of tumor cells was observed within the epidermis (**Figures 3A, B**). Immunohistochemical analysis confirmed melanocytic differentiation (positive for S100, HMB45, and Melan-A) and excluded epithelial malignancy (negative for CK5/6, CK7, CEA, EMA and Her-2) (**Figures 3C-J**). The Ki-67 proliferation index was 20%, consistent with aggressive biological behavior (**Figures 3K, L**). Finally, the patient was definitively diagnosed as SAM.

This case highlights the critical diagnostic challenges in SAM. The patient’s history of trauma, coupled with the presence of swelling and exudation, initially suggested a benign etiology. Importantly, the low clinical suspicion for SAM resulted in repeated misdiagnoses as nail infections and subsequent unnecessary nail avulsions—interventions that may potentially promote tumor dissemination. A definitive diagnosis was established based on histopathological and immunohistochemical analyses. Given the aggressive nature of the tumor and its digital location, which necessitated functional preservation, the patient was referred to the hand surgery department for definitive surgical management following multidisciplinary team evaluation. She subsequently underwent digit amputation. The clinical course of the patient is illustrated in **Figure 3**.

## Discussion

Melanoma, a malignant proliferation of melanocytes, classically manifests as pigmented lesions with heterogeneous coloration such as black, blue, or brown (7). Amelanotic melanoma (AM), a rare type of melanoma that lacks pigmentation, accounts for approximately 2% of all melanoma cases and about 25%-33% is observed in the subungual region (2, 3). AM is typically diagnosed in patients over 50 years of age, which is significantly older than the typical age at diagnosis for pigmented melanoma (8). However, in contrast to adults, approximately 70% of melanomas diagnosed in children are amelanotic (9). Although in general, subungual melanoma is more common in black Africans and Asians, it has been suggested that subungual amelanotic melanoma (SAM) occurs mainly in in White individuals with a predilection for the female gender (10–12).

SAM presents nonspecifically as nonpigmented lesion that originates from the nail bed, most commonly affecting the great toe and thumb (4). It present as pink, red, or flesh-colored papules or may manifest with longitudinal erythronychia (13). Common associated clinical features include onycholysis, notching, splitting, bleeding, or ulceration (14). Black pigmentation of the adjacent nail fold, known as Hutchinson’s sign, is often considered a diagnostic indicator of subungual melanoma. However, Hutchinson’s sign was negative in SAM (6). The clinical presentation of SAM presents a considerable diagnostic challenge, even for experienced dermatologists. SAM requires differentiation from the infectious dermatoses, immune-mediated disorders, other neoplastic conditions (2). Therefore, a delay in the diagnosis of a subungual amelanotic melanoma can occur due to its morphologic resemblance to other benign and malignant nail conditions, such as chronic onychia, onychomycosis, lichen planus and squamous carcinoma (5). Consequently, this often results in delayed diagnosis and, therefore, a worse prognosis compared to other forms of melanoma (15).

For initial screening, noninvasive diagnostic techniques such as dermoscopy are recommended. Dermoscopy enhances the ability to distinguish between benign and malignant nail lesions (16). Characteristic dermoscopic features of amelanotic melanoma include polymorphous vascular structures, such as milky-red areas, hairpin vessels, dotted vessels, and linear-irregular vessels. In addition to these vascular patterns, other dermoscopic criteria include scar-like depigmentation, rims of pigmentary networks, ulceration, and white lines, which represent the most significant nonvascular features of amelanotic melanoma (17). Dermoscopy is particularly valuable in the detection of amelanotic melanoma due to its ability to identify characteristic vascular patterns that compensate for the lack of melanin (18). Modern imaging modalities, such as positron emission tomography-computed tomography (PET-CT), is considered superior to conventional CT and MRI in evaluating amelanotic melanoma, as it enable the detection of primary melanoma approximately six months earlier than traditional imaging methods (19).

Although clinical examination, dermoscopy and PET-CT are valuable tools for identifying suspicious nail abnormalities, a definitive diagnosis of SAM necessitates histopathological evaluation following biopsy (20). Histopathological analysis typically reveals characteristic features of melanoma, including dermal infiltration by atypical melanocytes arranged in cords or nests (5). However, AM demonstrates substantial histopathological and cytological heterogeneity, which necessitates the use of immunohistochemical markers to ensure accurate diagnosis. The most widely utilized immunohistochemical markers include S100, Melan-A, HMB-45, MITF and Ki-67. Among these, S100 exhibits the highest sensitivity, whereas HMB-45, Melan-A and MITF demonstrate greater specificity (21). Notably, the intensity of HMB-45 staining is closely correlated with melanin content, thereby enhancing its specificity (2). Ki-67 serves as a valuable adjunct in distinguishing benign melanocytic proliferations from malignant lesions (22).

In this case, the middle finger lesion was initially diagnosed as chronic onychia following nail trauma at Hospital A. Over a period of two years, she underwent three nail avulsions and received systemic antibiotic therapy. Following the last avulsion, the nail failed to regrow. A comprehensive clinical and histopathological reassessment in our hospital confirmed a diagnosis of SAM. This case highlights the critical diagnostic challenges in SAM. SAM is a rare condition, and delayed diagnosis is frequently observed. One contributing factor to this diagnostic delay is that both patients and some unexperienced clinicians commonly atribute the lesion to traumatic injury as benign lesion (23). Importantly, the low clinical suspicion for SAM resulted in repeated misdiagnoses as nail infections and subsequent unnecessary nail avulsions—interventions that may potentially promote tumor dissemination. Unlike cutaneous melanoma, SAM is not commonly associated with ultraviolet radiation exposure due to the density of the nail plate, which substantially restricts light penetration (8). It has been proposed that trauma may play a role in the development of nail unit melanoma (24). Both acute and chronic trauma have been implicated. Acute trauma includes isolated traumatic events, while chronic trauma encompasses activities such as extensive manual labor, field plowing, and other physically demanding tasks often performed by rural women. Studies have reported that between 23% and 44% of patients recall experiencing trauma prior to the onset of subungual melanoma (25).It remains unclear whether this association arises from post-traumatic immunosuppression contributing to carcinogenesis.

Interestingly, a recent study found trauma to an subungual melanoma to be a a considerable risk factor in the carcinogenesis (24). Following trauma, the immune system triggers a cascade of inflammatory processes at the injury site, which is subsequently followed by a phase of localized inflammation resolution that supports tissue repair and remodeling (26). This localized immune response involves intricate interactions among resident immune cells, including macrophages and dendritic cells, soluble signaling molecules such as cytokines and chemokines, as well as recruited immune cells such as neutrophils, monocytes, and mesenchymal stromal cells (27). When these initial immune responses are sufficiently pronounced, they can lead to systemic effects, resulting in a condition known as post-traumatic immunosuppression (26, 27). Sterile trauma induces alterations in post-traumatic immune responses, potentially leading to a state of immune compromise in affected patients. When trauma is accompanied by infection, it may further modify the immune status (28). Persistent post-traumatic low-grade chronic inflammation within the nail could create a pro-tumorigenic microenvironment: mechanical disruption of the nail bed matrix may recruit macrophages and neutrophils, leading to the release of reactive oxygen species that directly damage melanocyte DNA and participate in cell proliferation (29).

Preliminary data suggests other prognostic indicators for subungual melanoma are similar to cutaneous melanomas (30). Investigations involving larger case series suggest that individuals with amelanotic melanoma often face significantly higher risks of mortality and recurrence, as well as lower 5-year survival and overall survival rates, compared to those with pigmented melanoma (2, 8). However, Moreau et al. found no significant difference in survival between amelanotic melanoma and pigmented melanoma at regional or distant stages (31). Furthermore, a large study reported that amelanosis did not demonstrate any prognostic significance after adjusting for established risk factors, including tumor stage, Breslow thickness, level of invasion, mitotic rate, and ulceration (2). Crucially, population-based research indicates that the worse prognosis associated with amelanotic melanoma stems entirely from more advanced disease stages at diagnosis, rather than from amelanosis itself (8). Therefore, the poorer outcomes observed in amelanotic melanoma are likely attributable to its association with advanced tumor stages, rather than representing an intrinsic risk factor (2, 8).

## Conclusion

In summary, SAM is often difficult to diagnose because it is rare and a great masquerader. This case underscores the importance of maintaining a high index of suspicion for SAM when evaluating atypical nail lesions. A low threshold for nail biopsy in cases of persistent prolonged swelling and exudation of a single nail is advised. Additionally, prior trauma to the nail may contribute to the development of SAM through post-traumatic immunosuppression and persistent low-grade chronic inflammation. However, the exact role of trauma in the pathogenesis of melanoma remains unclear and requires further investigation.

## Data Availability

The original contributions presented in the study are included in the article/**Supplementary Material**. Further inquiries can be directed to the corresponding author.
