# Supplementary material for: Rare subungual amelanotic melanoma presenting as prolonged swelling and exudation after trauma: case report and literature review
Source: Front Immunol. 2025 Sep 26;16:1661698. doi: 10.3389/fimmu.2025.1661698 (PMC12510839; doi:10.3389/fimmu.2025.1661698)
Supplement: Supplementary file 1 [file DataSheet1.pdf]

CARE
case report guidelines

CARE Checklist of information to include when writing a case report

(cc) BY-NC-ND

Topic	Item	Checklist item description	Reported on Line
**Title**	1	The diagnosis or intervention of primary focus followed by the words "case report"	Title
**Key Words**	2	2 to 5 key words that identify diagnoses or interventions in this case report, including "case report"	Key woeds
**Abstract (no references)**	3a	Introduction: What is unique about this case and what does it add to the scientific literature?	the fourth and the fifth sentences of the case presentation in abstract
	3b	Main symptoms and/or important clinical findings	the first and the second sentences of the case presentation in abstract
	3c	The main diagnoses, therapeutic interventions, and outcomes	the fifth,sentence of the case presentation in abstract
	3d	Conclusion—What is the main "take-away" lesson(s) from this case?	conclusion in abstract
**Introduction**	4	One or two paragraphs summarizing why this case is unique (**may include references**)	introduction
**Patient Information**	5a	De-identified patient specific information.	the first sentence of the case presentation
	5b	Primary concerns and symptoms of the patient.	the first paragraph of the case presentation
	5c	Medical, family, and psycho-social history including relevant genetic information	the last sentence of the of the first paragraph in case presentation
	5d	Relevant past interventions with outcomes	the fourth and sixth paragraph of the case presentation
**Clinical Findings**	6	Describe significant physical examination (PE) and important clinical findings.	the second paragraph of the case presentation
**Timeline**	7	Historical and current information from this episode of care organized as a timeline	the last paragraph in case presentation
**Diagnostic Assessment**	8a	Diagnostic testing (such as PE, laboratory testing, imaging, surveys).	the third, paragraphs of the case presentation
	8b	Diagnostic challenges (such as access to testing, financial, or cultural)	the fourth sentence of the of the third paragraph in case presentation
	8c	Diagnosis (including other diagnoses considered)	the last sentence of the of the third paragraph in case presentation
	8d	Prognosis (such as staging in oncology) where applicable	the fifth and the sixth sentences of the of the last paragraph in case presentation
**Therapeutic Intervention**	9a	Types of therapeutic intervention (such as pharmacologic, surgical, preventive, self-care)	the last paragraph in case presentation
	9b	Administration of therapeutic intervention (such as dosage, strength, duration)	the last paragraph in case presentation
	9c	Changes in therapeutic intervention (with rationale)	the fifth and the sixth sentences of the of the last paragraph in case presentation
**Follow-up and Outcomes**	10a	Clinician and patient-assessed outcomes (if available)	the sixth sentence of the of the last paragraph in case presentation
	10b	Important follow-up diagnostic and other test results	NA
	10c	Intervention adherence and tolerability (How was this assessed?)	NA
	10d	Adverse and unanticipated events	NA
**Discussion**	11a	A scientific discussion of the strengths AND limitations associated with this case report	The and the fifth paragraphs of the discussion
	11b	Discussion of the relevant medical literature **with references**.	The first to seven paragraphs of the discussion
	11c	The scientific rationale for any conclusions (including assessment of possible causes)	The first, second, third and the fourth paragraphs of the discussion
	11d	The primary "take-away" lessons of this case report (without references) in a one paragraph conclusion	the conclusion
**Patient Perspective**	12	The patient should share their perspective in one to two paragraphs on the treatment(s) they received	the fourth toseventh sentence of the first paragraph in case presentation
**Informed Consent**	13	Did the patient give informed consent? Please provide if requested	**Yes** ☑ **No** ☐
